# The epigenetic role of *ADRB3* DNA methylation in post-bariatric energy expenditure for women with obesity: a longitudinal observational study

**DOI:** 10.1038/s41598-026-46559-x

**Published:** 2026-03-31

**Authors:** Luísa Maria Diani, Lígia Moriguchi Watanabe, Natália Yumi Noronha, Guilherme da Silva Rodrigues, Marcela Augusta de Souza Pinhel, Chanachai Sae-Lee, Bruno Affonso Parenti de Oliveira, Carolina Ferreira Nicoletti, Gizela Pedroso Junqueira, Wilson Salgado, Júlio Sérgio Marchini, Carla Barbosa Nonino

**Affiliations:** 1 Department of Internal Medicine, Ribeirão Preto Medical School, University of São Paulo, Ribeirão Preto, São Paulo Brazil; 2 Department of Health Sciences, Ribeirão Preto Medical School, University of São Paulo, Ribeirão Preto, São Paulo Brazil; 3 Department of Statistics and Operational Research, Faculty of Sciences, University of Lisbon, Lisbon, Portugal; 4 Department of Obstetrics and Gynaecology, University Medical Center Groningen, Groningen, The Netherlands; 5 Ribeirão Preto School of Physical Education and Sport, University of São Paulo, São Paulo, Brazil; 6 Department of Molecular Biology, São José do Rio Preto Medical School, São José do Rio Preto, SP Brazil; 7 Research Division, Faculty of Medicine, Siriraj Hospital, Mahidol University, Bangkok, Thailand; 8 Applied Physiology and Nutrition Research Group, School of Physical Education and Sport, Faculdade de Medicina FMUSP, University of São Paulo, São Paulo, Brazil; 9 Center of Lifestyle Medicine, Faculdade de Medicina FMUSP, University of São Paulo, São Paulo, Brazil; 10 Rheumatology Division, Hospital das Clínicas HCFMUSP, Medical School, University of São Paulo, São Paulo, Brazil; 11 Department of Surgery and Anatomy, Ribeirão Preto Medical School, University of São Paulo, Ribeirão Preto, São Paulo, Brazil; 12 Department of Physical Education, State University of Minas Gerais, Divinópolis, Minas Gerais, Brazil

**Keywords:** Obesity, Bariatric surgery, Metabolic surgery, Epigenetics, DNA methylation, Energy metabolism, Biochemistry, Biomarkers, Diseases, Endocrinology, Molecular biology, Physiology

## Abstract

Bariatric surgery induces profound metabolic changes that may influence epigenetic mechanisms regulating genes involved in energy metabolism. Given the global burden of obesity on women’s health, understanding molecular responses to surgical treatment is essential. This study investigated the effect of Roux-en-Y gastric bypass (RYGB) on DNA methylation of the β-3 adrenergic receptor (*ADRB3*) gene and its influence on energy expenditure in women with obesity. Sixteen participants were evaluated before and six months after surgery, including measurements of body composition, resting metabolic rate (RMR), and metabolic biomarkers. After RYGB, significant reductions were observed in body weight, fat mass, glucose, cholesterol, triglycerides and absolute RMR. DNA methylation analysis revealed two differentially methylated CpG sites within *ADRB3*, and significant global hypomethylation. Postoperatively, *ADRB3* methylation correlated positively with RMR, explaining 38% of its variance, along with oxygen consumption and carbon dioxide production. Functional analysis supported *ADRB3*’s role in thermogenesis and lipid metabolism. These findings demonstrate that RYGB not only improves metabolic parameters, but also induces epigenetic remodeling of *ADRB3*, potentially influencing postoperative energy expenditure. This work provides new insights into the epigenetic regulation of metabolism and highlights *ADRB3* as a potential target for personalized obesity treatment in women.

**Supplementary Information:**

The online version contains supplementary material available at 10.1038/s41598-026-46559-x.

## Introduction

Obesity is a complex, multifactorial condition characterized by chronic dysregulation of energy homeostasis, resulting from interactions between genetic, environmental and behavioral factors^1^. Defined as the excessive accumulation of body fat, obesity has become an increasingly prevalent global health issue^2,3^. Data from the World Health Organization showed 1.9 billion adults with pre-obesity and 650 million adults with obesity around the world^4^, resulting in a substantial economic burden and escalating healthcare costs. The current costs of obesity, annually, are estimated at $2 trillion - nearly the equivalent of the costs of armed violence, war, and smoking combined^3^. In Brazil, the scenario is not different; the prevalence of obesity increased between 2006 and 2023, ranging from 11.8% to 24.3% in both sexes, but notably higher among women (12.1% to 24.8% in 2023)^5^. This demographic trend highlights the urgent need for targeted research and interventions focusing on women’s health in the context of obesity.

Obesity is also associated with significant health risks, affecting multiple physiological systems and contributing to the development of numerous comorbidities^6^. A wide range of clinical conditions is predisposed by obesity, including type 2 diabetes (T2DM), cardiovascular disease, and musculoskeletal disorders^7^, among more than 200 related conditions reported in the literature^8^. The growing prevalence of these comorbidities, along with the increasing rates of obesity, constitute a global health priority that requires effective responses for prevention and treatment^9^. For women, these risks can be further compounded by reproductive health issues, increased cancer risk, and specific psychosocial burdens, underscoring the critical need for effective weight management strategies tailored to their unique physiological and social contexts.

While the health risks associated with obesity highlight the urgency of effective interventions, understanding the mechanisms underlying weight regulation is essential to developing more successful treatment strategies^10^. Variations in body weight are associated with changes in Total Energy Expenditure (TEE), with three major components: the Thermal Effect of Food (TEF), corresponding to about 10–15% of TEE and defined as the increase of energy expenditure related to digestion, absorption, and storage of nutrients; Physical Activity Thermogenesis (PAT), that could increase the metabolic rate with formal or informal physical activities; and the Resting Metabolic Rate (RMR), the main component of TEE, responsible for around 60–75% of total daily expended energy^11^. Given its important contribution to TEE, RMR represents a key determinant of energy balance and a relevant predictor of metabolic adaptation during weight loss interventions^11^. Resting metabolic rate can be assessed by Indirect Calorimetry (IC), a method based on thermodynamic principles that estimates energy expenditure from measurements of oxygen consumption and carbon dioxide production^12^.

Lifestyle modifications such as nutritional education and intervention, physical exercise, and behavior changes, sometimes associated with pharmacotherapy, constitute the first-line treatment for weight reduction^13^. Still, in many cases, they are insufficient to achieve significant and sustainable results^14^. Bariatric surgery is an option for patients with obesity when non-surgical weight loss methods have been unsuccessful^15^. Surgical eligibility criteria include a Body Mass Index (BMI) ≥ 40 kg/m^2^ without coexisting comorbidities or BMI ≥ 35 kg/m^2^ and one or more obesity-related complications treatable by weight loss^16^. The Roux-en-Y gastric bypass technique is a bariatric procedure that involves creating a small proximal gastric pouch, separating it from the remainder of the stomach, which is then connected to the jejunum by a gastrojejunostomy^17^. Bariatric surgery induces profound physiologic adaptations, including reduced food intake and altered hormone secretion contributing to weight loss and maintenance^18^. For this reason, bariatric surgery represents a unique human model to investigate systemic metabolic and epigenetic remodeling associated with substantial and sustained weight loss.

Beyond weight reduction, surgical treatment can lead to several beneficial health effects, including improvements in obesity-related comorbidities^19^. However, these outcomes vary among individuals, as many metabolic alterations occur due to an interaction between environment, lifestyle, and genetic factors^20^. In this context, epigenetic mechanisms have gained attention as potential mediators of interindividual variability in metabolic adaptation following surgical weight loss^21^. Epigenetics refers to heritable changes in gene regulation, characterized as changes to chromatin structure and function without altering the DNA sequence^22^. Recent evidence has demonstrated that epigenetic regulation may mediate the beneficial effect induced by surgery modifying the epigenome, mainly through alterations in the DNA methylation profile^23–26^.

DNA methylation, an important epigenetic mechanism involving a direct chemical modification to DNA, consists of the biochemical addition of a methyl group that occurs mainly in CpG dinucleotides through the action of methyltransferase enzymes (DNMTs). These enzymes transfer a methyl group from S-adenyl methionine (SAM) to the fifth carbon of a cytosine residue forming 5-methylcytosine (5mC)^27^. This process has been associated with regulating body weight by controlling the expression of specific genes, as it is involved in weight-related functions, including appetite, adiposity, adipogenesis, glucose, and lipid metabolism^28^. Importantly, DNA methylation may modulate metabolic pathways by altering gene responsiveness to hormonal and neural signals rather than directly affecting protein structure or function^28^.

Energy metabolism is coordinated by signaling pathways that regulate lipolysis, thermogenesis and energy expenditure^29^. Epigenetic regulation has been shown to affect genes involved in signal transduction and transcriptional control, thereby modulating the sensitivity and capacity of metabolic pathways to respond to environmental and physiological stimuli^30^. Particular attention has been given to genes involved in energy expenditure and thermogenic pathways^29–31^. Among these, the β-3 adrenergic receptor gene (*ADRB3*) plays an important role in mediating catecholamine-induced lipolysis in white and brown adipose tissue, facilitating the release of free fatty acids for thermogenesis and contributing to the regulation of body weight in humans^32^. Given its upstream regulatory role in energy expenditure pathways, *ADRB3* represents a biologically plausible epigenetic target to investigate metabolic adaptations following bariatric surgery^33–35^. While the methylation assay is genome-wide, the present analysis was hypothesis-driven and focused on *ADRB3* and a predefined panel of energy metabolism-related candidate genes. Despite its established physiological importance, the contribution of *ADRB3* DNA methylation to obesity-related metabolic adaptations, particularly following bariatric surgery, remains insufficiently characterized, especially in women^36,37^.

Therefore, the present study aimed to evaluate the impact of bariatric surgery on the DNA methylation pattern of the *ADRB3* gene and to investigate whether these epigenetic changes are associated with energy expenditure in women with obesity. In addition, DNA methylation patterns of other key genes involved in energy metabolism were explored to contextualize *ADRB3*-related findings. Focusing on women addresses a clinically relevant population while acknowledging that sex-specific comparisons were beyond the scope of this study.

## Methods

Sixteen women with obesity were enrolled in this study and evaluated both before and six months after Roux-en-Y gastric bypass surgery, with follow-up conducted at the Hospital das Clinicas, Faculty of Medicine of Ribeirao Preto, University of São Paulo (HC/FMRP-USP). All participants received standardized postoperative nutritional supplementation according to current clinical guidelines, including routine multivitamin supplementation. Individual folate (folic acid) status was not measured in this cohort, and therefore could not be included as a covariate in the statistical models.

Exclusion criteria included any modifications to the standard surgical technique (DGYR), loss to follow-up, pregnancy, lactation during the study period, and the presence of comorbidities that could affect energy expenditure, such as diabetes, hyperthyroidism, gastrointestinal disorders, chronic infections, or cancer. The exclusion of pregnancy and lactation was particularly relevant given the female-only cohort, ensuring hormonal stability during the study period. The study protocol was approved by the Institutional Research Ethics Committee (CAAE: 62874122.8.0000.5440) in accordance with the ethical guidelines set out in the Declaration of Helsinki.

### Anthropometric evaluation and body composition

Body weight was measured using a Filizola^®^ scale with a precision of 0.2 kg, and height was measured with a vertical stadiometer to an accuracy of 0.5 cm. Body mass index (BMI) was calculated from the measured weight and height. Abdominal circumference (AC) was measured at the largest circumference around the umbilical scar using a non-elastic measuring tape with a 0.1 mm resolution. Body composition was assessed by bioelectrical impedance analysis using the Quantum BIA 450 Q device (RJL System). Resistance and reactance values were entered into validated equations specific for obese women^38^ to determine fat-free mass (FFM). Fat mass (FM) was calculated as the difference between total body weight and FFM. The use of validated equations specific for obese women enhances the accuracy and relevance of body composition data within this all-female cohort.

### Resting metabolic rate (RMR)

RMR was measured via indirect calorimetry, assessing oxygen consumption (O_2_) and carbon dioxide production (CO_2_) using the QUARK-RMR device (COSMED, Rome, Italy). The device was automatically calibrated prior to each assessment using standard gas concentrations. Participants were instructed to fast for six hours, refrain from physical activity, and avoid coffee or black tea for 24 h before testing. A low-flow face mask (Canopy, 0–50 L/min; COSMED) was used during assessment. Subjects remained awake, lying supine in a quiet, dimly lit, temperature-controlled room (21–24 °C), with evaluations performed in the morning between 8:00 and 10:00. Oxygen consumption (VO_2_) and carbon dioxide production (VCO_2_) were recorded for 30 min, discarding the first 10 min to ensure metabolic stabilization^39^. RMR was calculated using the Weir equation:

$${\text{RMR (kcal/day)}}=3.941\times {\mathrm{VO}}2 {\mathrm{(L/min)}}+1.106\times {\text{VCO2 (L/min)}}.$$

### Blood sample collection

Blood samples were collected after a 12-hour overnight fast. Whole blood was processed immediately after collection. Biochemical parameters, including glycemia and lipid profile, were assessed according to standard laboratory protocols at HC/FMRP-USP. Total cholesterol (TC), triglycerides (TG), LDL cholesterol (LDL-c), and HDL cholesterol (HDL-c) were determined by automated colorimetric methods, and fasting glycemia was measured enzymatically. For genetic analyses, genomic DNA was isolated from peripheral whole blood.

### DNA methylation analysis

DNA Extraction: Genomic DNA was extracted from peripheral blood using the Illustra Blood Genomic Prep Mini Spin kit (GE HealthCare), following the manufacturer’s instructions.

Assessment of DNA Integrity: DNA concentration and purity were assessed spectrophotometrically (NanoDrop 2000c), measuring absorbance at 260 nm and the 260/280 nm ratio, with a minimum quality threshold set at 1.8. DNA quantity was further verified using the PicoGreen DNA Quantification Reagent (Invitrogen, Carlsbad, CA, USA).

Sodium Bisulfite Treatment: A total of 500 ng of DNA was treated with sodium bisulfite using the EZ DNA Methylation Kit (Zymo Research, CA, USA), as recommended by the manufacturer. This process converts unmethylated cytosines to uracils, while methylated cytosines remain unchanged.

DNA Methylation Array: Genome-wide methylation analysis was performed using the Infinium HumanMethylation450k BeadChip platform (Illumina, San Diego, CA). Bisulfite-converted DNA was amplified and hybridized to the BeadChip, and fluorescence intensity was detected using the Illumina iScanSQ platform. Data were extracted with Genome Studio Methylation software (v1.9.0, Illumina).

### Bioinformatics analysis

#### Data extraction and preprocessing

Data analysis was performed using RStudio (version 3.6.2) and the ChAMP package (version 2.24.0, Bioconductor)^40^. Nutritional, biochemical, calorimetric, and demographic data were entered into a Samplesheet for subsequent analysis. Raw data from the iScan system underwent quality control, normalization, and cell fraction correction in accordance with established protocols^40^. The resulting matrix comprised normalized beta values representing the methylation percentage at each CpG site^41,42^.

#### Selection of targets

Using the Illumina manifest (Infinium HumanMethylation450k v1.2), 15 CpG sites within the *ADRB3 *gene were identified. Beta values for these target sites were extracted from the normalized matrix for further analysis. Beta values were transformed into M-values, the log_2_ ratio of the intensities of methylated to unmethylated probes^43^, as M-values provide greater statistical reliability for differential methylation analysis due to improved homoscedasticity compared to beta values. Although the array provides genome-wide coverage, the present work followed a hypothesis-driven candidate-gene strategy focused on energy expenditure pathways. In addition to *ADRB3,* DNA methylation data were explored for selected candidate genes involved in energy expenditure and metabolic regulation, including *UCP1, UCP2, UCP3, PLIN1, PPARG2* and *GNAS,* using CpG sites annotated in the Illumina manifest.

#### Functional enrichment analysis

The *ADRB3* gene symbol was submitted to the STRING database (Protein-Protein Interaction Networks Functional Enrichment Analysis) for KEGG pathway enrichment analysis^44^.

### Statistical analysis

Data are presented as mean ± standard deviation (SD) with corresponding p-values. The Shapiro-Wilk test was used to assess normality of the data. Differences before and after bariatric surgery were analyzed using either the paired t-test or the Wilcoxon signed-rank test, as appropriate. Correlations were evaluated using Pearson or Spearman coefficients. Statistical analyses were conducted using SPSS 14.0 (SPSS Inc., Chicago, IL, USA) and GraphPad Prism (GraphPad Software Inc., San Diego, CA). Statistical significance was defined as *p* < 0.05. Given the hypothesis-driven candidate-gene approach, correction for multiple testing was not applied. We acknowledge that false discovery rate (FDR) control is required for discovery-driven epigenome-wide analyses; therefore, results should be interpreted within the context of a targeted candidate-gene strategy.

## Results

The study cohort consisted of sixteen women with obesity, with a mean age of 37 ± 10.4 years (Table 1). Following bariatric surgery, significant reductions were observed in several clinical parameters. There was a notable decrease in body weight (23.2%; *p* < 0.01), abdominal circumference (AC) (14%; *p* < 0.01), and body mass index (BMI) (22.9%; *p* < 0.01), resulting in a reclassification of participants from grade III to grade I obesity. In terms of body composition, both fat-free mass (FFM) (11.7%; *p* < 0.01) and fat mass (FM), measured in kilograms (36.7%; *p* < 0.01) and percentage (18.2%; *p* < 0.01), were significantly reduced. The substantial weight loss, primarily attributable to the reduction in fat mass, led to a significant relative increase in the proportion of fat-free mass (13.4%; *p* < 0.01).

**Table 1:** Participants’ clinical characteristics. (*n* = 16)

	Preoperative	Postoperative	*p*-value
**Anthropometry**			
Age (years)	37 ± 10,4		
Height (m)	1.63 ± 0.08		
Weight (kg)	119.6 ± 17.5	91.8 ± 13.4	< 0.01
AC (cm)	127.8 ± 13.8	109.9 ± 13.2	< 0.01
BMI (kg/m^2^)	44.9 ± 6.6	34.6 ± 5.4	< 0.01
**Body composition**			
FFM (kg)	64.1 ± 6.8	56.6 ± 5.8	< 0.01
FFM (%)	53.9 ± 3.7	62.3 ± 5.3	< 0.01
FM (kg)	55.6 ± 11.7	35.2 ± 9.1	< 0.01
FM (%)	46.1 ± 3.7	37.7 ± 5.3	< 0.01
**Biochemical indicators**			
Glycemia (mg/dL)	92.1 ± 14.1	83.7 ± 6.2	0.02
Total Cholesterol (mg/dL)	181.1 ± 34.1	158.7 ± 24.4	0.01
HDL-c (mg/dL)	43.4 ± 8.5	44.95 ± 8.9	0.55
LDL-c (mg/dL)	109.7 ± 29.7	94.5 ± 19.9	0.07
Triglycerides (mg/dL)	134.8 ± 41.1	84.7 ± 19.9	< 0.01
**Indirect calorimetry**			
VO_2_ (l/min)	0.31 ± 0.04	0.28 ± 0.03	< 0.01
VCO_2_ (l/min)^†^	0.22 ± 0.02	0.19 ± 0.02	< 0.01
RMR (kcal/day)	2136.9 ± 287.2	1870.8 ± 227.8	< 0.01
Adjusted RMR			
RMR/weight (kcal/kg/day)	18 ± 2.1	20.5 ± 1.9	< 0.01
RMR/FFM (kcal/kg/day)	33.4 ± 3	33.1 ± 3.1	0.76

Values are expressed as mean ± standard deviation. ^†^Shapiro-Wilk test *p* > 0.05. Paired Student’s t-test for parametric and Wilcoxon test for non-parametric samples, *p* < 0.05.

*BMI* body mass index, *AC* abdominal circumference, *FFM* fat-free mass, *FM* fat mass, *VO*_*2*_ consumed oxygen volume, *VCO*_*2*_ produced carbon dioxide volume, *RMR* resting metabolic rate.

In addition to improvements in anthropometric measures, participants demonstrated significant enhancements in biochemical markers commonly associated with obesity-related comorbidities. After surgical intervention, there were significant decreases in fasting glycemia (*p* = 0.02), total cholesterol (*p* = 0.01), and triglycerides (*p* < 0.01). No significant changes were observed in HDL-cholesterol (*p* = 0.55) or LDL-cholesterol (*p* = 0.07). It is noteworthy that prior to surgery, participants’ biochemical parameters were not clinically altered, consistent with the study’s inclusion and exclusion criteria that required metabolic stability.

Analysis of resting metabolic rate (RMR) via indirect calorimetry revealed a significant reduction in absolute RMR (12.4%; *p* < 0.01), as well as decreases in oxygen consumption (VO_2_) (9.6%; *p* < 0.01) and carbon dioxide production (VCO_2_) (13.6%; *p* < 0.01). As energy expenditure is closely related to body size and composition, RMR was further evaluated relative to both total body weight and fat-free mass to assess metabolic efficiency and account for individual variability. The RMR adjusted for body weight increased after surgery (12.2%; *p* < 0.01), while no significant difference was observed in the RMR adjusted for fat-free mass (0.9%; *p* = 0.76).

Bioinformatics analysis was conducted using RStudio and the ChAMP package from Bioconductor. The beta values for the 15 CpG sites within the *ADRB3* gene were extracted from the whole normalized matrix and transformed into M-values (log_2_ ratio of methylated to unmethylated signal intensity) to assess differential DNA methylation levels. Differential methylation analysis of these CpG sites before and after bariatric surgery revealed that two sites, cg00432461 and cg03103178, were differentially methylated six months postoperatively: cg00432461 was hypomethylated at the TSS1500 region, while cg03103178 was hypermethylated in the IGR region (Table 2). No significant differences were detected in the M-values of other CpG sites following the surgical intervention.

**Table 2:** Characteristics of the CpG sites from the *ADRB3* gene (*n* = 16).

Illumina ID	CHR	Gene location	Mean M values preoperative		Mean M values postoperative		*p*-value
cg10357888	8	TSS1500	− 1.34	± 0.29	− 1.41	± 0.27	0.09
cg19806221	8	TSS1500	− 0.2	± 0.35	− 0.12	± 0.33	0.06
**cg00432461**	**8**	**TSS1500**	**4.07**	**± 0.55**	**3.69**	**± 0.33**	0.02*
cg01386493	8	TSS1500	0.59	± 0.35	0.55	± 0.32	0.35
cg02174634	8	TSS200	− 1.65	± 0.28	− 1.7	± 0.29	0.39
cg23460057	8	TSS200	− 1.37	± 0,0.9	− 1.43	± 0.20	0.30
cg05182393	8	Body	3.72	± 0.39	3.73	± 0.48	0.95
cg09258813	8	1stExon	− 3.91	± 0.22	− 4.1	± 0.37	0.12
cg09442828	8	1stExon	− 1.59	± 0.22	− 1.62	± 0.28	0.45
cg27498114	8	1stExon	− 4.95	± 0.42	− 5.04	± 0.43	0.48
cg16223863	8	1stExon	− 3.8	± 0.23	− 3.94	± 0.17	0.08
cg17619823^†^	8	1stExon	− 4.01	± 0.43	− 4.02	± 0.26	0.59
cg22275864	8	Body	− 2.17	± 0.23	− 2.32	± 0.28	0.07
**cg03103178** ^†^	**8**	**IGR**	− **1.8**	**± 0.26**	− **1.89**	**± 0.32**	0.05*
cg24438644	8	IGR	− 2.58	± 0.24	− 2.58	± 0.26	0.99

Note: Values are expressed as mean ± standard deviation. ^†^Shapiro-Wilk test *p* < 0.05. *Paired student’s t-test for parametric and Wilcoxon test for non-parametric samples (*p* < 0.05). CHR: Chromosome; TSS: Transcription Start Site; IGR: Intergenic Region.

**Fig. 1:**
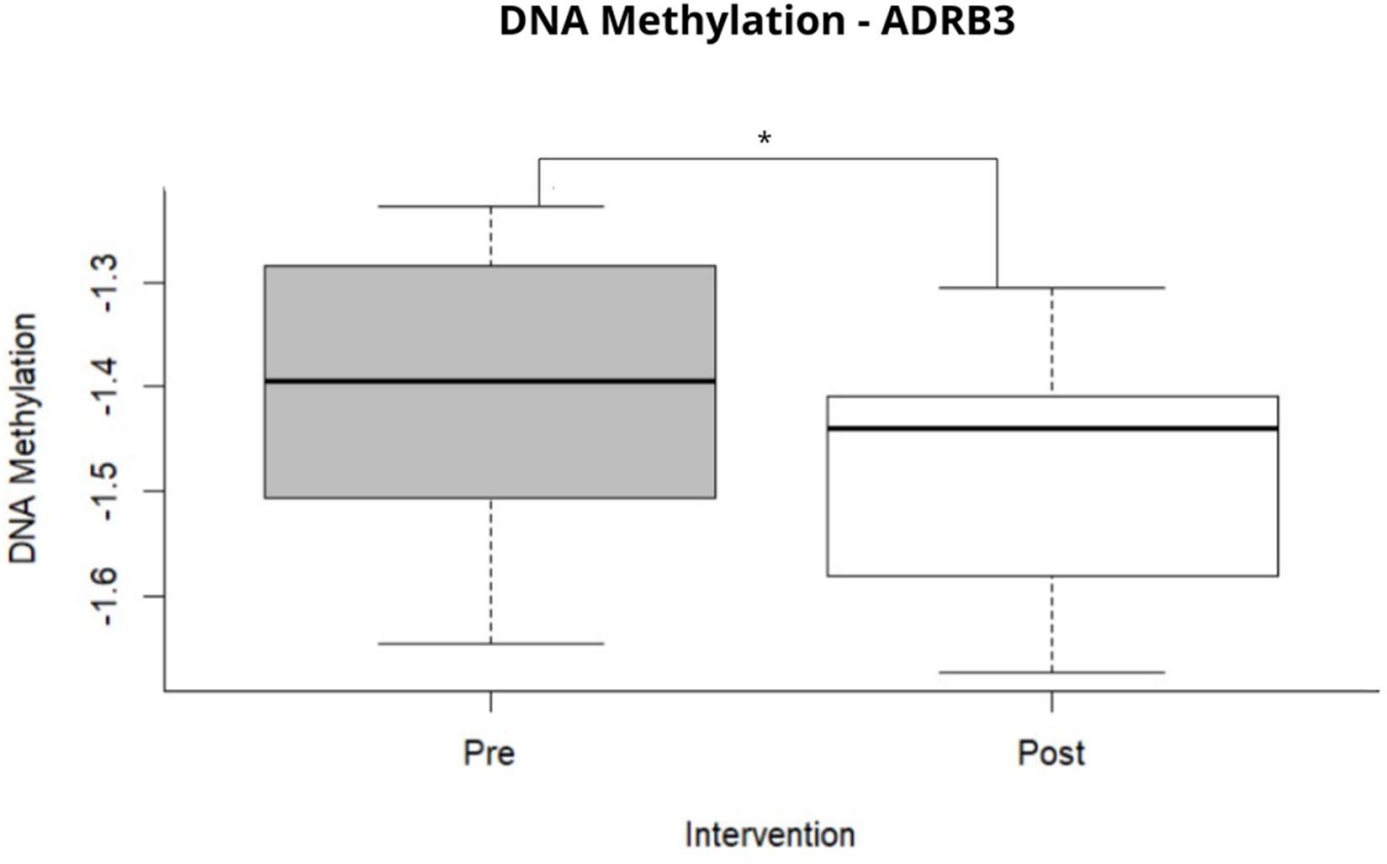
Average CpG sites of the *ADRB3* gene before and after Roux-en-Y gastric bypass. Note: *Paired student’s t-test for parametric samples (*p* < 0.05). Pre: preoperative period; Post: postoperative period.

Global DNA methylation levels of the *ADRB3* gene were assessed by calculating the average of its M-values across all probes targeting this gene. Paired Student’s t-test identified significant hypomethylation of *ADRB3* following surgical treatment (preoperative: − 1.39 ± 0.12; postoperative: − 1.48 ± 0.11; *p* = 0.0183) (Fig. 1). In addition to *ADRB3*, DNA methylation data obtained from the methylation array were explored for selected genes involved in energy expenditure and metabolic regulation, including *UCP1*,* UCP2*,* UCP3*,* PLIN1*, *PPARG2* and *GNAS* (Supplementary Table 1). Across the CpG sites evaluated for these candidate genes, we did not observe statistically significant or consistent pre-to-post changes at 6 months, supporting the specificity of the *ADRB3* signal within the candidate panel.

**Fig. 2:**
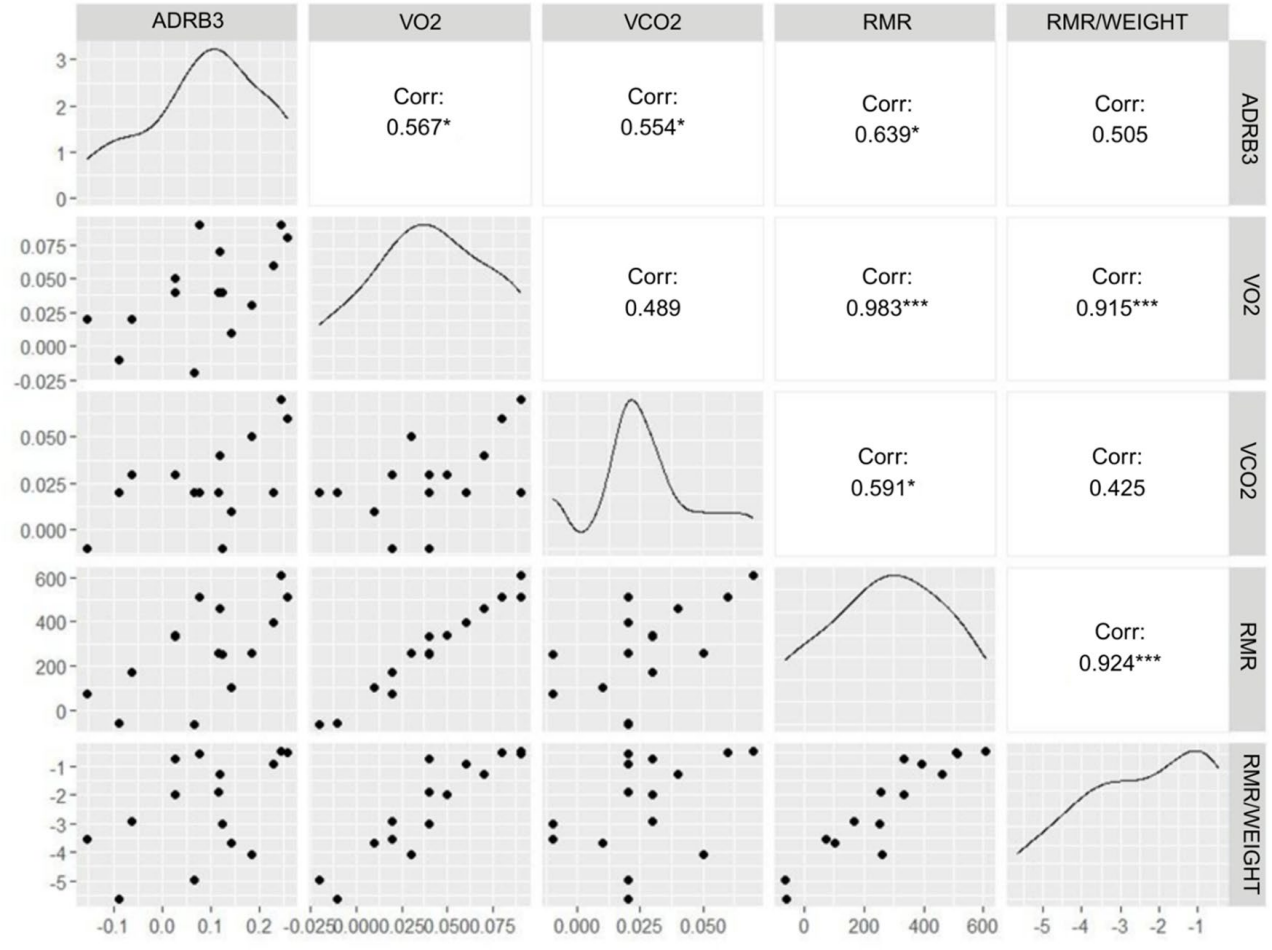
Correlation between the variation in methylation M-values from the *ADRB3* gene with the difference between the pre- and post-bariatric surgery periods of the IC variables of women submitted to bariatric surgery after six months (*n* = 16). Note: Pearson’s coefficient analysis. Values are represented as “r” coefficient. *p < 0.05 **p < 0.01; ***p < 0.001. *ADRB3*: beta-adrenergic receptor 3; VO2: volume of oxygen consumed; VCO2: volume of carbon dioxide produced; RMR: resting metabolic rate; RMR/weight: resting metabolic rate corrected for weight.

Correlation analysis using Pearson’s coefficient was performed to explore the relationship between *ADRB3* gene methylation (M-values) and parameters derived from indirect calorimetry. After surgery, statistically significant positive correlations were identified between *ADRB3* methylation and VO_2_ (*r* = 0.567, *p* < 0.05), VCO_2_ (*r* = 0.554, *p* < 0.05), and RMR (*r* = 0.639, *p* < 0.05). No significant correlation was detected between *ADRB3* methylation and RMR adjusted for body weight (*r* = 0.505, *p* > 0.05) (Fig. 2). These associations support a potential functional relevance that epigenetic modifications in *ADRB3* are intricately linked to changes in energy expenditure following bariatric surgery in women, suggesting a direct molecular pathway influencing metabolic adaptation.

To determine whether post-surgical *ADRB3* methylation contributed to changes in energy expenditure characteristics, independent linear regression analyses were performed (Table 3). The results indicated that *ADRB3* DNA methylation accounted for a significant proportion of the variance in VO_2_ (R^2^ = 0.29; *p* = 0.0181), VCO_2_ (R^2^ = 0.22; *p* = 0.0351), RMR (R^2^ = 0.38; *p* = 0.0059), and RMR adjusted for weight (R^2^ = 0.23; *p* = 0.0325) following surgery, with the strongest association observed for RMR (explaining 38% of the variance). This predictive power underscores the clinical relevance of *ADRB3* methylation as an epigenetic biomarker for metabolic outcomes in women post-bariatric surgery.

**Table 3:** Independent Linear regression analysis shows changes in *ADRB3* DNA methylation contribution to changes in energy expenditure traits after surgical treatment.

Model	*R* ^2^	β	*p*
ΔVO_*2*_ ~ ΔMethylation	0.29	2.67	0.02
ΔVCO_*2*_ ~ ΔMethylation	0.22	2.33	0.03
ΔRMR ~ ΔMethylation	0.38	3.23	0.01
ΔRMR/Weight ~ ΔMethylation	0.23	2.37	0.03

Note - ΔMethylation is the variation in ADRB3 DNA methylation before and after bariatric surgery.

**Fig. 3:**
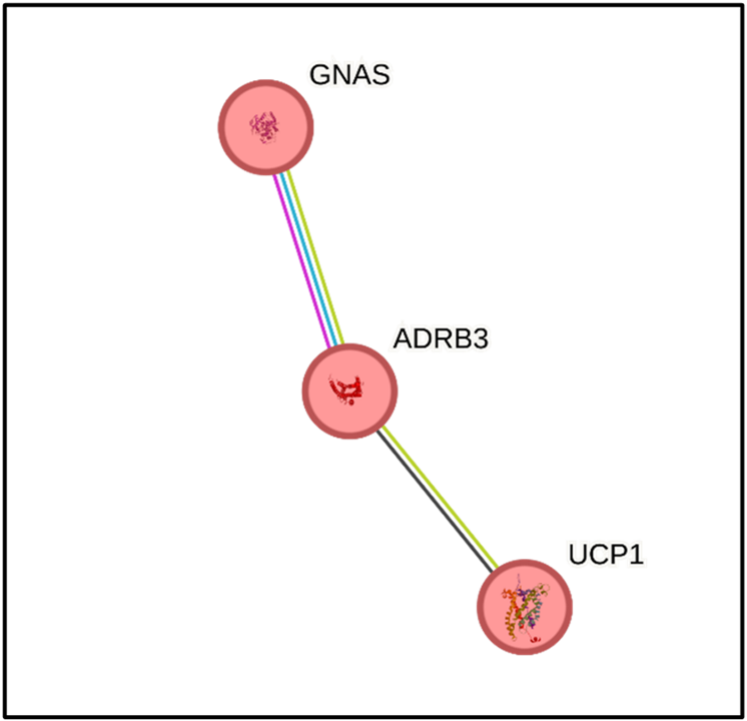
Gene interactions of the *ADRB3* gene in the thermogenesis pathway. Note: Nodes in red: genes involved in the regulation of thermogenesis. The lines represent interactions between genes. Blue line: known database interactions. Purple line: experimentally determined interactions. Green line: neighboring genes. Yellow line: interactions cited in the same text. Black line: co-expression. *ADRB3*: beta-adrenergic receptor 3; *GNAS:* guanine nucleotide-binding G protein, XLas isoform alpha subunit;* UCP1*: uncoupling protein 1.

Functional enrichment analysis using the STRING database identified biological processes and pathways associated with the *ADRB3* gene. The findings indicated that *ADRB3* is involved in key pathways relevant to obesity and weight loss, notably the regulation of lipolysis in adipocytes (FDR = 0.0040) and thermogenesis (FDR = 0.00052). These pathways are of particular significance to the present study. In this cohort, *ADRB3* methylation may influence resting metabolic rate. Protein-protein interaction analysis further confirmed *ADRB3’s* central role in thermogenesis, demonstrating interactions with other genes implicated in this pathway, including guanine nucleotide-binding protein *GNAS* (XLas isoform alpha subunit) and uncoupling protein 1 (*UCP1*). The interaction between *ADRB3* and *GNAS* was supported by both known database and experimentally derived data, while the interaction between *ADRB3* and *UCP1* was based on co-expression data (Fig. 3). These functional insights strongly support the biological plausibility of *ADRB3* epigenetic modifications driving metabolic improvements in women post-RYGB.

## Discussion

The findings of the present study demonstrate a significant reduction in anthropometric variables, including weight, BMI, and abdominal circumference six months after bariatric surgery in a cohort of women with obesity. On average, participants changed from grade III to grade I obesity postoperatively. Additionally, significant reductions were observed in body composition parameters such as fat-free mass (FFM, kg) and fat mass (FM, both kg and %), along with an increase in the relative percentage of FFM. These improvements are clinically relevant in women with obesity, in whom excess adiposity is associated with increased risk of reproductive complications and certain cancers; however, the present study was not designed to test sex-specific effects. These results are consistent with those of Felipe, et al. (2023), who reported significant reductions in weight and BMI following gastric bypass at six months postoperative^45^. Other studies evaluating patients undergoing bariatric surgery have also reported a significant decrease in anthropometric indices and body composition^46–48^. Bariatric surgery is widely recognized as the most effective intervention for severe obesity, reducing the prevalence and associated comorbidities. Regardless of the surgical modalities, significant weight loss is expected and typically achieved, contributing to numerous health benefits for these individuals^19^. For women, achieving substantial weight loss and improvements in body composition can have far-reaching positive impacts, including enhanced reproductive health outcomes, reduced risk of obesity-related cancers, and improved quality of life.

During the initial months post-bariatric surgery, the combination of reduced food intake, loss of FFM, and low physical activity levels contributes to a decline in resting metabolic rate (RMR), a challenge for sustaining long-term weight loss^49,50^. Consistent with previous findings by ArgyraKopoulou et al. (2023)^51^, Cardia et al. (2023)^49^, Reichmann et al. (2023)^52^, and Fidilio et al. (2021)^53^, our study demonstrates a significant reduction in absolute RMR six months postoperative — a decrease expected primarily to the loss of FFM, particularly in the early postoperative period^46^. Despite the decline in absolute RMR, an increase in weight-adjusted RMR was observed, while RMR normalized to FFM remained unchanged. This pattern is reported in the literature^46,54^ and the study by REICHMANN, M. et al. (2023) demonstrated a sustained elevation in weight-adjusted RMR up to two years postoperatively^52^. Although loss of FFM and consequent reduction in RMR may contribute to weight regain, an increased weight-adjusted RMR may be a positive prognostic indicator, particularly in women^49^. Understanding these RMR adaptations is critical for developing tailored nutritional and exercise interventions that can support sustainable weight loss and prevent weight regain in female patients.

Understanding epigenetic regulation requires a focus on the DNA methylation status of CpG sites. In this study, differential methylation analysis identified two differentially methylated CpG sites in the *ADRB3* gene, one in a promoter region (TSS1500) and another in an intergenic region (IGR). The assessment of site-specific DNA methylation provides insight into the epigenetic environment of individual genes, which provides important information about regulation and expression levels. It is recognized that the functional consequences of DNA methylation depend on the location of the affected CpG site^55^. Using the M-value matrix, the average methylation status of *ADRB3* was determined, revealing significant hypomethylation of *ADRB3* following surgical intervention. This observation of global hypomethylation of *ADRB3* in women post-RYGB is a novel observation, suggesting a broad epigenetic reprogramming that may underpin the observed metabolic improvements.

Given *ADRB3’s* crucial role in pathways that regulate energy homeostasis, we assessed the relationship between postoperative changes in *ADRB3* methylation and parameters measured by indirect calorimetry (IC), specifically to determine the association with energy expenditure in these women. DNA methylation patterns of *ADRB3* postoperatively were significantly associated with VO_2_, VCO_2_, and RMR, but not with weight-adjusted RMR. Although site-specific differences may be modest in absolute terms in array-based methylation studies, the observed *ADRB3 *changes were accompanied by consistent associations with energy expenditure variables (VO_2_, VCO_2_ and RMR). Independent linear regression analysis further confirmed that *ADRB3* methylation contributed to the variance of these IC variables after surgery, with RMR explaining 38% of *ADRB3* DNA methylation variation post-treatment. These robust associations between *ADRB3 *methylation and key energy expenditure metrics provide strong evidence for the functional importance of this epigenetic modification in mediating metabolic adaptations following bariatric surgery in women.

It is important to highlight that, to our knowledge, no previous studies have evaluated *ADRB3* DNA methylation in peripheral blood six months after bariatric surgery specifically in a female cohort. Limited work has explored ADRB3 methylation in obesity: Lima et al. (2019) reported hypermethylation of* ADRB3* in women with obesity, which correlated with total cholesterol, LDL-c, and triglycerides; Guay et al. (2014) noted hypermethylation in men developing obesity, associated with LDL-c, suggesting *ADRB3* as a candidate gene for dyslipidemia. Oliveira et al. (2018), studying eutrophic and overweight individuals, found no difference in *ADRB3* methylation between groups, but did not assess severe obesity^36,56,57^. While previous studies have reported *ADRB3* hypermethylation in obesity, our findings indicate hypomethylation after weight loss intervention, consistent with existing literature that links hypomethylation of certain metabolic genes to improved metabolic function. The observed shift from hypermethylation (in obesity) to hypomethylation (post-weight loss) in *ADRB3* provides a dynamic epigenetic signature of metabolic recovery in women.

Functional enrichment analysis identified genes that interact with *ADRB3* in metabolic pathways. In association with the *GNAS* gene, *ADRB3* may regulate lipolysis in white adipose tissue, by hydrolyzing triacylglycerols into fatty acids and glycerol for energy use^58^. ADRB3 is also implicated in the regulation of thermogenesis, an essential process for maintaining body temperature, ensuring that cellular and physiological functions occur in different environmental conditions^36,59^. Thermogenesis is mainly controlled by norepinephrine, which is released in the sympathetic nervous system in response to cold or dietary stimuli, activating ADRB3 and GNAS in brown adipose tissue. Adrenergic receptors bind to G protein, activating adenylate cyclase (AC), to increase cyclic adenosine monophosphate (cAMP) and activate protein kinase A (PKA), leading to the cAMP-PKA signaling pathway. PKA phosphorylates target proteins and a subset of transcription factors to promote the expression of uncoupling protein 1 (UCP1), encoded by the *UCP1* gene. Finally, UCP1 is responsible for the process where chemical energy is converted into heat in adipocytes, expected to improve overweight and obesity conditions^60^. Consistent with these pathways, the study conducted by Sánchez, E. et al. evaluated DNA methylation remodeling in adipose tissue in 41 women after six months of bariatric surgery. They reported the *GNAS* complex locus as one of the genes differentially methylated with higher alterations after surgery, presenting a significant correlation with BMI changes, Total Cholesterol, and LDL-c profiles^61^. In contrast, the present study did not observe significant changes in *GNAS* DNA methylation following bariatric surgery. This discrepancy may reflect differences in sample size, analytical approaches, tissue specificity, or interindividual variability in epigenetic responses to surgical weight loss. These findings highlight the complexity of epigenetic regulation in energy metabolism and the importance of tissue-specific analyses.

A hypothesis to explain the obesity state, besides leptin or insulin resistance, is Catecholamine resistance - the inability of adipocytes from individuals with obesity to properly respond to catecholamines. This mechanism may predict future weight gain in some patients, and the initiating step in catecholamine signaling is β-adrenergic receptors (β3-AR) activation on the cell membrane^62^. Valentine et al., 2022, demonstrate that, in vivo and in vitro, catecholamine resistance occurs with a significant reduction in the expression of Adrb3 mRNA and β3-AR protein. The author suggests that β-adrenergic receptor downregulation is the key molecular mechanism in catecholamine resistance^58^, which results in a reduction of thermogenesis and lipolysis^62^. Our finding of *ADRB3* hypomethylation postoperative may be consistent with mechanisms involved in improved catecholamine responsiveness, leading to improved thermogenesis and lipid metabolism, contributing significantly to the sustained weight loss observed in these women.

In addition to the epigenetic findings, obesity is frequently associated with metabolic alterations, including dyslipidemia, characterized by hypercholesterolemia, hypertriglyceridemia, low HDL-c, and elevated LDL-c and type 2 diabetes mellitus, with perturbations in glucose and insulin metabolism^47,63^. In the present study, significant reductions were found in fasting glycemia, total cholesterol, and triglyceride levels, though no significant changes were observed in LDL-c or HDL-c. A meta-analysis by Carswell et al. (2016) investigating the effects of RYGB on lipid profiles found significant reductions in total cholesterol, LDL-c, and triglyceride levels three months after surgery^63^. The RYGB procedure involves the creation of a small gastric pouch connected to the jejunum in a Roux-Y configuration, which leads to reduced secretion of pancreatic enzymes and gastric acid. This results in delayed digestion, decreased lipid emulsification, and reduced absorption in the ileum, leading to changes in lipid and glucose metabolism^45,63^. These anatomical and physiological adaptations reinforce findings from additional studies^47,48,64^ and highlight the potential for metabolic improvement and remission of comorbidities such as dyslipidemia and T2DM following significant weight loss due to surgical treatment^45^. These metabolic improvements are particularly salient in women, as dyslipidemia and insulin resistance are major contributors to cardiovascular disease risk, which is a leading cause of mortality in women globally.

A primary limitation of this study is the small sample size. In addition, the absence of a male control group limits direct comparisons between sexes and limits the interpretation of sex-specific effects of bariatric surgery on DNA methylation and energy expenditure. Although the study focused exclusively on women, reflecting their predominance among bariatric surgery patients and specific hormonal considerations, future studies including both sexes are needed to directly assess related differences in epigenetic and metabolic responses. Finally, because our cohort included only women and no male comparison group, we could not assess sex-specific differences in *ADRB3* methylation or energy expenditure adaptations after RYGB; therefore, our findings should be interpreted as specific to this female cohort. Another limitation is the use of peripheral blood for DNA methylation analysis, given that *ADRB3* functions predominantly in adipose tissue. Nevertheless, peripheral blood is a metabolically responsive tissue and may reflect systemic inflammatory and vascular consequences of obesity. Future investigations should aim to validate these findings in adipose tissue samples and explore potential tissue and sex-specific epigenetic mechanisms. Nutritional biomarkers were not assessed, particularly folate levels, which are known to influence DNA methylation. Although all participants followed a standardized postoperative multivitamin supplementation protocol, individual folate status was not directly measured and therefore cannot be excluded as a potential confounding factor.

Despite these limitations, the present study provides novel insights into the association between *ADRB3* DNA methylation, energy expenditure, and weight loss in women following bariatric surgery.

## Conclusion

Surgical treatment for obesity resulted in significant changes in DNA methylation and energy expenditure among women with grade III obesity six months after the procedure. Participants experienced substantial metabolic improvements, evidenced by reductions in anthropometric measures (weight, BMI, and abdominal circumference), body composition (FM in kg and %, FFM in kg), and biochemical indicators (glycemia, total cholesterol, and triglycerides), supporting both the efficacy of bariatric surgery for weight loss and its positive impact on metabolic health and quality of life in female patients. RMR, particularly when adjusted for body weight, also showed favorable changes postoperatively, attributable primarily to the marked loss of FFM, a key determinant of resting metabolic rate in this population.

Crucially, the *ADRB3 *gene, essential for the regulation of lipolysis and thermogenesis, was found to be significantly hypomethylated and associated with RMR after bariatric surgery in this female cohort. These findings underscore the multifactorial nature of both the development and treatment of obesity and, more importantly, highlight a novel epigenetic mechanism, specifically involving *ADRB3 *DNA methylation, that contributes to the beneficial metabolic adaptations observed in women following RYGB. This study provides critical insights into the molecular underpinnings of weight loss success in women and lays the groundwork for further exploration of epigenetic profiles in identifying predictors of surgical outcomes and developing sex-specific therapeutic strategies for obesity.

## Supplementary Information

Below is the link to the electronic supplementary material.

Supplementary Material 1

## Data Availability

The Datasets generated during the current study are available from the corresponding author on reasonable request.

## Abbreviations

RYGB: Roux-en-Y gastric bypass
ADRB3: β-3 adrenergic receptor
RMR: Resting metabolic rate
BMI: Body mass index
AC: Abdominal circumference
FFM: Fat-free mass
FM: Fat mass
T2DM: Type 2 diabetes mellitus
TEE: Total energy expenditure
TEF: Thermal effect of food
PAT: Physical activity thermogenesis
IC: Indirect calorimetry
VO_2_: Oxygen consumption
VCO_2_: Carbon dioxide production
DNMTs: DNA methyltransferase enzymes
SAM: S-adenyl methionine
5mC: 5-methylcytosine
GNAS: Guanine nucleotide-binding G protein, XLas isoform alpha subunit
HC/FMRP-USP: Hospital das Clínicas, Faculty of Medicine of Ribeirão Preto, University of São Paulo
TC: Total cholesterol
TG: Triglycerides
LDL-c: Low-density lipoprotein cholesterol
HDL-c: High-density lipoprotein cholesterol
TSS1500: Transcription Start Site (1500 base pairs upstream)
IGR: Intergenic region
UCP1: Uncoupling protein 1
β3-AR: β3-adrenergic receptors
AC: Adenylate cyclase
cAMP: Cyclic adenosine monophosphate
PKA: Protein kinase A
FDR: False discovery rate
CHR: Chromosome
SD: Standard deviation
