# Supplementary material for: The epigenetic role of ADRB3 DNA methylation in post-bariatric energy expenditure for women with obesity: a longitudinal observational study
Source: Sci Rep. 2026 Mar 31;16:15555. doi: 10.1038/s41598-026-46559-x (PMC13186948; doi:10.1038/s41598-026-46559-x)
Supplement: Supplementary file 1 — Supplementary Material 1 [file 41598_2026_46559_MOESM1_ESM.docx]

| **Supplementary Table 1.** Comparison of average CpG site methylation levels of candidate genes before and after Roux-en-Y gastric bypass. | | | |
| --- | --- | --- | --- |
| **Gene** | **Preoperative** | **Postoperative** | ***p-value*** |
| ***UCP1*** | -2.71 ± 0.19 | -2.81 ± 0.25 | 0.1625 |
| ***UCP2*** | -2.32 ± 0.09 | -2.33 ±0.09 | 0.7811 |
| ***UCP3**** | -2.24 ± 0.12 | 2.24 ±0.15 | 0.4037 |
| ***PLIN1*** | 0.11 ± 0.1 | 0.14 ±0.13 | 0.4418 |
| ***PPARG2*** | -1.25 ± 0.13 | -1.32 ±0.11 | 0.088 |
| ***GNAS*** | -0.26 ± 0.11 | -0.30 ±0.12 | 0.3755 |
| *Results are presented as mean ± standard deviation. * Shapiro–Wilk normality test p < 0.05. Paired Student’s* t *test or Wilcoxon signed-rank test; significance set at p < 0.05. UCPs: uncoupling proteins (1, 2, and 3); PLIN1: perilipin 1; PPARG2: peroxisome proliferator-activated receptor gamma 2; GNAS: guanine nucleotide-binding G protein, XLas isoform alpha subunit;* | | | |
